# Inflamed Microglia like Macrophages in the Central Nervous System of Prodromal Parkinson’s Disease

**DOI:** 10.1101/2025.05.16.654530

**Published:** 2025-05-21

**Authors:** Le Zhang, Yoshiaki Yasumizu, M. Elizabeth Deerhake, Jeonghyeon Moon, Nicholas Buitrago-Pocasangre, Anthony Russo, Haowei Wang, Biqing Zhu, John P. Seibyl, Vijaya Reddy, Qianchang Wang, Maria Grazia Spillantini, David A. Posner, Menna Clatworthy, Tomokazu S. Sumida, Erin E. Longbrake, Jesse M. Cedarbaum, David A. Hafler

**Affiliations:** 1. Department of Neurology, Yale School of Medicine, New Haven, CT, 06510, USA.; 2. Department of Neuroscience, Yale School of Medicine, New Haven, CT, 06510, USA.; 3. Aligning Science Across Parkinson’s (ASAP) Collaborative Research Network, Chevy Chase, MD 20815, USA.; 4. Department of Immunobiology, Yale School of Medicine, New Haven, CT, 06510, USA.; 5. Department of Biostatistics, Yale School of Public Health, New Haven, CT, 06510, USA.; 6. Program of Computational Biology and Bioinformatics, Yale University, New Haven, CT 06510, USA.; 7. Molecular Immunity Unit, University of Cambridge Department of Medicine, Cambridge, UK; 8. Department of Clinical Neurosciences, University of Cambridge Department of Medicine, Cambridge, UK; 9. Broad Institute of MIT and Harvard, Cambridge, MA, 02142, USA.

## Abstract

We investigated the role of inflammation in the pathogenesis of prodromal Parkinson’s Disease (PD), performing single-cell RNAseq analysis of cerebrospinal fluid (CSF) and blood from 111 individuals, comparing control subjects with early prodromal PD and later PD to patients with multiple sclerosis (MS). Surprisingly, we identified a pleocytosis in the CSF, most pronounced in patients with early PD. Single-cell RNAseq revealed increases in CSF-specific microglia-like macrophages expressing JAK-STAT and TNFα signaling signatures in prodromal PD, with a lack of T cell activation in the CSF. The CSF macrophages exhibited similar transcriptional profiles to dural macrophages from human α-synuclein-expressing PD model mice. These findings uncover a myeloid-mediated TNFα inflammatory process in the CNS of patients with prodromal PD, suggesting a novel pathological mechanism in disease etiology.

## Introduction

Inflammation with activation of both the adaptive and innate immune system is critical in mediating tissue repair ^1^. While some degree of inflammation is observed in virtually all central nervous system (CNS) diseases representing responses to tissue injury, primary T cell mediated autoimmunity such as Multiple Sclerosis (MS) can be a primary cause of CNS inflammation. Parkinson’s Disease (PD) is a neurodegenerative disorder, predominantly in older populations, characterized by the progressive loss of dopaminergic neurons and the accumulation of α-synuclein aggregates in the brain. Both genetic and pathologic evidence support the notion that CNS inflammation contributes to PD pathogenesis ^2–6^. Specifically, the presence of TNFα in microglia and the recent discovery of autoreactive α-synuclein T cells in early PD ^7^ raises the hypothesis that autoreactive T cells drive early CNS inflammation in prodromal PD, as is seen in relapsing-remitting MS, with later, secondary neurodegeneration manifesting as classic PD ^8–13^.

We recently identified myeloid cells in the cerebrospinal fluid (CSF) expressing microglia-associated cell surface proteins, such as APOE, C1Q, and TREM2, that are presumably derived from a monocyte lineage. While first observed in higher numbers in the CSF of patients with HIV^14^, they are also observed in the CSF of healthy subjects. These CSF microglia-like macrophages share gene expression patterns with border-associated macrophages located along the brain vasculature at the interface of the brain parenchyma ^15,16^. Surprisingly, the frequency of this population of microglia-like macrophages was markedly decreased in the CSF of patients with autoimmune, relapsing-remitting MS as compared to age-matched healthy controls ^17^. With highly effective B cell depletion therapy, this population re-emerged in the CSF of MS patients, with associated increases in myeloid TNFα expression in both CSF and blood, suggesting an important role in autoimmune disease pathogenesis ^17^.

One early clinical manifestation of α-synucleinopathy is Rapid Eye Movement Sleep Behavior Disorder (RBD), a parasomnia that can appear years or even decades before the onset of typical symptoms of PD or Dementia with Lewy Bodies (DLB) ^18^. Thus, RBD serves as an early indicator of future α-synuclein-related neurodegeneration. Additionally, there is strong epidemiologic data indicating a link between autoimmune disorders, such as inflammatory bowel disease and PD, adding further support for a prominent role for the immune system in mediating the inception and progression PD pathology ^19,20^. Thus, examining whether there is CNS inflammation in RBD as a manifestation of prodromal synucleinopathy and determining the pathways mediating inflammation can provide a rationale for clinical experiments to prevent the disease and to provide more convincing evidence that an early autoimmune process proceeds a secondary neurodegenerative form of disease.

Here, we performed single-cell RNAseq analysis of CSF and blood from 84 individuals, including 34 with RBD, 18 with PD without RBD, 15 PD and RBD, 15 age-matched healthy controls, and 29 patients with recent onset MS. We unexpectedly identified a lymphocytic pleocytosis in the CSF of patients with RBD, that was not present in patients with longer standing PD, similar to what is observed in patients with autoimmune MS. However, in marked contrast to MS, single-cell RNAseq in prodromal PD revealed an increase in the frequency and cell numbers of CSF microglia-like macrophages expressing IL6-JAK-STAT3 and TNFα signaling pathways. This inflammatory signature was shared with that of brain microglia of PD patients and CNS-dural sinus myeloid cells and border-associated macrophages in PD model mice expressing human truncated α-synuclein in dopaminergic neurons. These findings reveal a myeloid-mediated TNFα inflammatory process in the CNS during the prodromal stage of PD, suggesting a newly described pathologic process in disease etiology.

## Results

### Single-cell immune atlas of PBMCs and CSF in prodromal and early stage of PD

To profile the immunological changes in prodromal and across PD stages, we generated a single-cell RNAseq immune atlas from paired peripheral blood mononuclear cells (PBMCs) and CSF cells. A total of 84 participants were enrolled, of whom 73 contributed blood and CSF samples suitable for analysis and categorized into four groups: RBD (n=36), PD without RBD (described as PD; n=15), PD with RBD (described as PD-RBD; n=18), and age-matched healthy controls (HC; n=15) (Table S1,2). Clinical and demographic information was collected, and PBMCs and CSF samples were obtained from all participants. The demographic and clinical characteristics of our study population were similar to other cohorts (Table S1). The mean ages of the subjects in the four groups ranged from 64 to 89 years; 77% were males. Of the RBD group, 19 (53%) were hyposmic, as defined by an UPSIT score below the 15^th^ percentile for age and gender ^21^, 7 (19%) evidenced reduced dopamine transporter (DaT) binding in the striatum, and 25 (69%) showed Synuclein Aggregating Activity (SAA) in their CSF. In all, 26 (72%) of the RBD subjects met MDS Prodromal PD Likelihood Ratio criteria for prodromal PD ^22^, and were thus defined as a “high-risk” population. Of the subjects who entered with a diagnosis of RBD, six were diagnosed with either PD or Lewy Body Dementia (LBD) during the course of the study.

**Figure 1:**
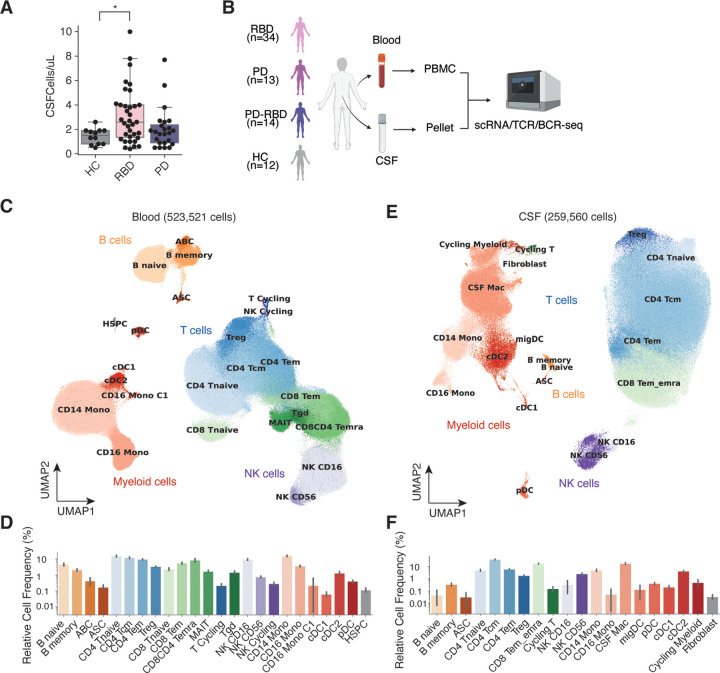
Single-cell immune atlas of PBMCs and CSF in prodromal and early stage of PD. (A) CSF cell counts. (HC: 1.4 ± 0.7; RBD: 3.3 + 2.3, PD: ?? cells/uL; p = 0.045, ANOVA followed by Tukey-Kramer post-hoc test) (B) Schematic overview of the project workflow. (C-F) Uniform Manifold Approximation and Projection (UMAP) plots of single-cell data (C,E) and corresponding cell frequencies (D,F) for blood (C,D) and CSF (E,F). Error bars indicate 95% confidence intervals (D,F).

We first examined the CSF for evidence of inflammation by quantitative analysis of approximately ~30 mL of CSF from each subject. Unexpectedly, subjects with RBD showed an increase of CSF cells compared to age-matched HC subjects (3.3 + 2.3 vs 1.4 ± 0.7 cells/uL; p = 0.045, ANOVA followed by Tukey-Kramer post-hoc test; Fig. 1A), indicative of inflammation in the CNS of prodromal PD patients. We then performed droplet-based single-cell RNAseq with VDJ sequencing using the 10x Genomics Chromium platform. After stringent quality control, 523,521 cells from PBMCs (73 donors) and 259,560 cells from CSF (72 donors) were retained (Fig. 1B–F, Fig. S1A). The average number of genes and percentage of mitochondrial genes detected per cell across all samples were 1,833 genes and 2.67% in PBMCs and 1,929 genes and 1.83% in CSF, respectively, reflecting high-quality single-cell transcriptomic data across both sample types. Based on the clusters defined by unsupervised clustering, we identified major immune cell types: B cells, myeloid cells, T cells, and NK cells in both PBMCs and CSF. Subclusters within these groups were further annotated based on known marker genes, resulting in 24 clusters in PBMCs and 20 clusters in CSF (Fig. 1D–G, Fig. S1B,C). The cell cluster annotations were validated using CellTypist ^23^ (Fig. S2E,F).

In PBMCs, the proportions of immune cells were as follows: B cells 8.09%, myeloid cells 20.8%, T cells 59.6%, and NK cells 11.0% (Fig. 1D). In contrast, CSF showed distinctly different proportions consistent with our previous findings ^24^: B cells 0.53%, myeloid cells 23.8%, T cells 73.0%, and NK cells 2.59% (Fig. 1F). Compared to PBMCs, CSF had a higher proportion of T cells and myeloid cells, and a lower proportion of B cells and NK cells. At the subcluster level, specific differences were observed. For example, populations such as CD4 T naive cells (15.9% in PBMC, 5.62% in CSF), CD16^+^ monocytes (3.56% in PBMC, 0.09% in CSF), and CD16^+^ NK cells (10.0% in PBMC, 0.34% in CSF) were less abundant in CSF, while clusters such as cDC2 (1.06% in PBMC, 3.98% in CSF) and CD4 T central memory (Tcm) cells (12.3% in PBMC, 41.6% in CSF) were enriched. As we previously reported^14^, microglia-like macrophages in the CSF (termed CSF Mac), which expressed *C1QC* and *TREM2* specifically, were the most abundant myeloid cell population in CSF and were uniquely observed in this compartment. These findings suggest that, while the CSF immune environment largely consists of circulating immune cells similar to those in PBMCs, it exhibits significant differences in cell composition. The CSF likely represents a unique immune microenvironment linked to the CNS and CNS borders. Overall, we successfully constructed a single-cell immune cell atlas of PBMCs and CSF in RBD and PD.

### Pronounced immunological changes in prodromal PD

**Figure 2:**
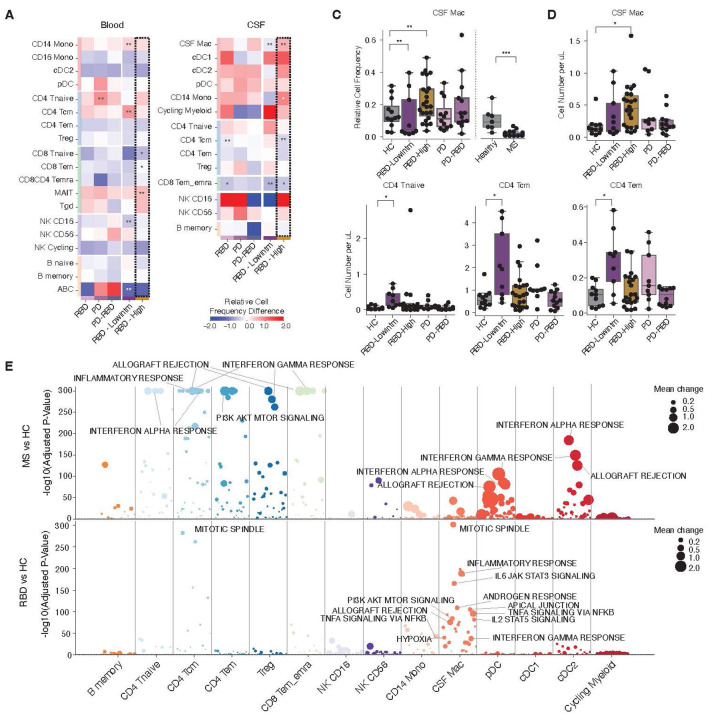
Global characterization highlighted activation of CSF Macrophages in prodromal PD. (A,B) Cell population changes for RBD, PD, and PD-RBD were analyzed in Blood (A) and CSF (B) using scCODA ^25^ (a Bayesian statistical tool) relative to HC, with RBD stratified by PD risk score (right heatmaps). See materials and methods for the details. FDR thresholds: 0.05, ‘**’; 0.1, ‘*’. Color indicates fold change in relative cell frequency. The RBD high-risk group is outlined with dotted lines. (C) CSF macrophages exhibited significant alterations, shown by per-patient frequencies (right). The frequencies in MS ^17^ and age-matched HC are also shown (left). For PD cohort, the scCODA statistical significance is marked similarly with (A,B). For MS, Statistical testing was performed using the Mann–Whitney U test for each cell population, followed by multiple testing corrections. The adjusted p-value was 1.12 × 10^-4^. (D) The absolute cell number of CSF Mac was and T cell populations calculated by the proportion of the cell from scRNAseq and the cell count data. (E) Hallmark gene sets enriched in MS (upper) and RBD (lower) across cell types in comparison to HC (materials and methods). Only positively associated gene sets were visualized. The dashed line at the bottom indicates Padj = 0.05. See Fig. S2 for other comparisons. Only positively associated gene sets were visualized. The dashed line at the bottom indicates Padj = 0.05. (A,B,E lower) Panels include only clusters containing >1000 cells for blood and >500 cells for CSF. (E upper) The same cell types with (F) were retained for the visualization.

To identify PD-specific immune alterations, we analyzed our single-cell immune atlas to assess both quantitative changes (shifts in cell frequencies and absolute cell numbers) and qualitative changes (alterations in gene expression). To evaluate changes in cell frequencies, we used a Bayesian model ^25^ to compare RBD, PD, and PD-RBD groups against age-matched healthy controls (Fig. 2A–C). RBD exhibits heterogeneity in features such as hyposmia and CSF SAA status, both of which are known to be associated with PD onset ^26^. Using these factors, we further stratified RBD individuals based on predicted PD risk ^22^. This analysis revealed a significant increase in the CSF Mac population in the RBD high-risk group (Fig. 2B,C). Additionally, the absolute numbers of CSF Mac, CD4^+^ Tnaive, Tcm, and Tem cells in CSF were increased as compared to healthy controls (Fig. 2D) while there was a decrease in the relative proportion of CD4^+^ Tcm cells and CD8^+^ T effector memory/terminally differentiated effector memory (em/emra) cells in the RBD group (FDR < 0.05 and < 0.1 respectively). As previously described ^27^, we observed an increase in CD4^+^ T naive cells in the peripheral blood of prodromal PD (FDR < 0.05).

We next examined gene expression changes within each cluster (Fig. S2A). Compared to PBMCs, we observed more pronounced gene expression changes in CSF, especially in CD4^+^ T cells, CD14^+^ monocytes, and the CSF Mac population. Notably, when stratifying RBD patients by MDS Prodromal PD probability score, the RBD high-probability group exhibited the highest number of differentially expressed genes (DEGs). These findings suggest that both cell frequency and gene expression changes are most pronounced in the RBD high-probability group, with relatively smaller differences observed in PD and PD-RBD. Furthermore, in CSF, CD4^+^ T cells and CSF Mac demonstrated coordinated changes in both cell numbers and gene expression.

To interpret these gene expression changes, we calculated scores for each cell population across conditions using Hallmark gene sets from the Molecular Signatures Database (MSigDB) (Fig. 2E, S2B–F, Table S3,4). In PBMCs, oxidative phosphorylation was broadly upregulated in T cells from RBD and PD-RBD patients, suggesting that peripheral circulating T cells maintain a homeostatic state, consistent with the observed increase in naive CD4^+^ T cells. In CSF, the most pronounced gene program changes were detected in myeloid populations, particularly in CSF Mac. In the RBD group, significant enrichment of pathways such as mitotic spindle (Mean change = 0.640, padj=4.33 × 10^−302^), inflammatory response (Mean change = 0.689, padj=2.45 × 10^−189^), IL6-JAK-STAT3 signaling (Mean change = 0.447, padj=3.97 × 10^−166^), and TNFα signaling via NF-κB (Mean change = 0.807, padj=2.84 × 10^−96^), were observed. These findings suggest heightened cellular activity and immune responses in the CSF Mac population in prodromal PD.

### Inflammatory signature in MS versus RBD CSFs

**Figure 3:**
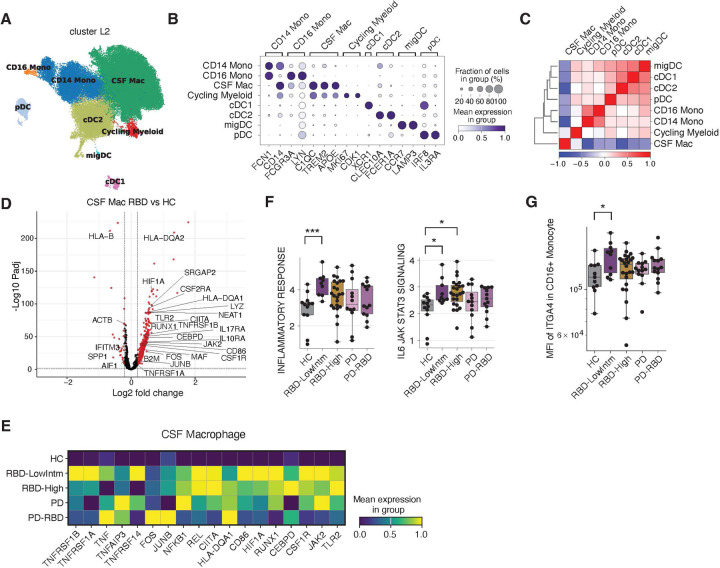
CSF macrophages activation and linked gene patterns with PD brain microglia. (A) Myeloid cell populations in CSF on UMAP embeddings. (B) Dot plot depicting signature genes’ mean expression levels and percentage of cells expressing them across clusters. Marker genes for the plot were manually selected. (C) Heatmap showing the transcriptome correlation between myeloid cell populations. (D) Volcano plot showing the differential expressed genes in CSF Macrophage (CSF Mac) between RBD vs HC. For the visualization, genes with mean expression > 0.5 were selected. Genes with FDR < 0.1, Log2 fold change > 0.2 or < −0.2 were highlighted in red. (E) Heatmap showing scaled mean expression of the representative genes. The value was scaled for each gene. (F) Sample-wise HALLMARK gene set activity. *: FDR < 0.05. (G) Mean Fluorescence Intensity (MFI) distribution of IGTA4 in CD16^+^ Monocytes. (p=0.0147)

A major paradox in the field of autoimmunity revolves around the TNFα pathway, presumably relating to the different genetic architectures of human autoimmune diseases ^28,29^. Specifically, while anti-TNFα therapy is an effective treatment for Inflammatory Bowel Disease (IBD) and rheumatoid arthritis (RA), it paradoxically induces exacerbations in patients with MS^30,31^. Moreover, three independent studies have shown a highly significant decrease in the incidence of PD in IBD patients on anti-TNFα therapy with an increased concordance of IBD and PD ^19,20^. Based on these observations, we hypothesized that the CSF Mac population in prodromal PD would be characterized by elevated TNFα signaling and exhibit a distinct gene expression profile compared to MS. We analyzed single-cell dataset of CSF cells from 29 MS patients and 7 age-matched healthy individuals. As recently reported in a different MS dataset ^17^, we observed a marked reduction in the CSF Mac population that was confirmed with a recent meta-analysis of other MS datasets (Fig. 3C) ^16,17,32^. Gene set enrichment analysis revealed a pattern opposite to that observed in RBD, with strong enrichment of gene sets in T cells, B cells, and dendritic cells, while enrichment in CSF macrophages was minimal (Fig. 3E, Table S5). Notably, TNFα signaling via NF-_κ_B showed a negative change in MS CSF Mac (Mean change=−0.716, padj=4.75 × 10^−5^). Furthermore, we applied (1) a detailed analysis pipeline specialized for circulating CD4^+^ T cells ^33,34^ and (2) TCR-based estimation of T cell activity ^35^. Both approaches consistently suggested that T cells are not activated in PD or RBD (Supplementary Note). These findings suggest that the regulatory mechanisms governing CSF Mac population differ between MS and PD, particularly in the context of TNFα signaling pathway.

### Inflammatory signature in CSF Macrophages

We conducted a detailed analysis of CSF Mac, which exhibited notable quantitative and qualitative changes. As previously described, CSF Mac population shares transcriptomic similarity with microglia and expresses macrophage markers, such as *C1QC*, *APOE*, and *TREM2,* at higher levels compared to other myeloid populations (Fig. 3A–C) ^14,16,17^. Previous studies also suggested this population resembles border-associated macrophages ^15,16^. To further analyze the tissue specificity of this CSF Mac population and its similarity with microglia, we integrated myeloid populations from both PBMCs and CSF with publicly available datasets of microglia ^36^ and cross-tissue myeloid cells ^23^, comparing their expression profiles (Fig. S3). While the analysis as expected revealed that CSF Mac population was transcriptionally close to microglia and tissue-resident macrophages, distinct differences were also observed. For instance, *SPP1*, *P2RY12*, and *TMEM119* were more highly expressed in microglia than in CSF Mac (Fig. S3). These findings highlight both the similarities and the divergence in expression patterns between CSF Mac and microglia. We next compared the gene expression of CSF Mac between controls and RBD. The analysis revealed upregulation of HLA class II molecules and their regulator *CIITA*, as well as increased expression of *HIF1A*, mitochondrial genes, AP1 family genes, *JAK2*, *TLR2*, and *TNFRSF1A* and *TNFRSF1B* in RBD (Fig. 3D,E, Table S6). These changes were consistent with hallmark gene set analyses, which showed elevated scores for pathways such as inflammatory response and IL6-JAK-STAT3 signaling in RBD patients (Fig. 3F). Both RNA seq and flow cytometry analysis revealed an increase in integrin alpha 4 subunit (ITGA4), a key molecule for CNS homing, in CD16^+^ monocytes from the blood of RBD low-risk individuals, suggesting that monocyte migration to the CNS is more active during the prodromal phase of PD (Fig. 3G). This finding suggests that the activation of myeloid cells in peripheral blood is reflected later than in the CSF.

We further hypothesized that CSF Mac might be functionally linked to microglial changes occurring in the brains of PD patients. To test this hypothesis, we utilized publicly available single-nucleus RNAseq data profiling multiple brain regions from 100 donors, including 75 PD cases ^37^. The analysis revealed that genes upregulated in CSF Mac in RBD were also significantly upregulated in the primary motor cortex and prefrontal cortex of PD brains (Fig. S4A,B). These results suggest that CSF Mac are not only activated during the prodromal phase of PD but also reflect microglial changes occurring within the PD brain. In addition, we attempted to examine myeloid populations in the CNS border region. As dural tissue was not available, we examined dural myeloid cells from PD model mice expressing aggregation-prone truncated human α-synuclein under the control of the tyrosine hydroxylase promoter ^38^ (material and methods). The dural macrophage expressed CSF Mac marker genes such as *Trem2*, *Apoe*, and *C1qc* (Fig. S4C). We found that TNFα related genes and MHC class II genes were upregulated in PD model mice, consistent with the changes observed in CSF Mac in prodromal PD. These data suggest that synchronized gene expression changes occur in brain parenchymal microglia, CNS-broader macrophage, and CSF Mac in prodromal and clinical stages of PD.

### Enhanced cell-cell communication in the immune landscape of RBD

**Figure 4:**
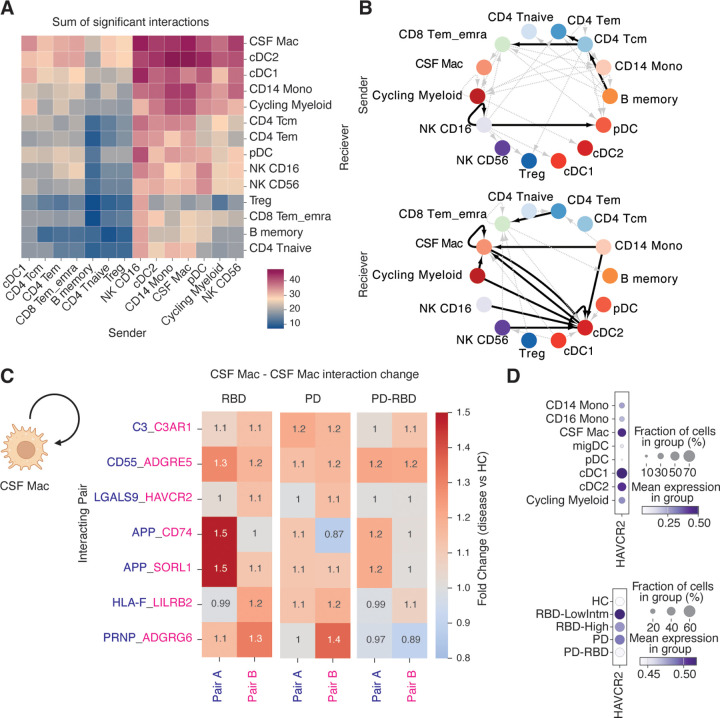
Cell-cell interaction analysis showed enhanced receiver activity in CSF myeloid cell populations (A) The heatmap showing the number of interactions inferred by CellphoneDB ^39^. The x-axis shows the sender, and the y-axis shows the receiver. (B) The activated ligand-receptor pairs in RBD compared to HC (materials and methods). The upregulated sender signals (upper) and receiver signals (lower) are shown. Interactions with FDR < 0.2 are shown in the black lines. Potentially upregulated interactions (p < 0.05) were shown in the dashed grey lines. (C) Heatmaps show fold change in disease conditions compared to HC for representative CSF Mac ligand-receptor pairs. (D) Dot plot showing the expression of HAVCR2 across CSF myeloid cell populations (upper) and in CSF Mac population across conditions (lower).

Next, we examined cell-cell interactions in the CSF using CellPhoneDB^39^. The ligand—receptor interaction prediction suggested that myeloid cells, including CSF Mac and cDC2, would exhibit robust ligand—receptor signals and that there would be more interactions between lymphocytes and myeloid cells than among lymphocytes alone (Fig. 4A). Focusing on the RBD group, which observed more pronounced immunological changes, we investigated cell-cell interaction pairs with substantial shifts in signaling dynamics. We found that ligands associated with signaling were moderately elevated (FDR<0.2) in lymphocyte subsets such as CD4 Tcm, NK CD16, and memory B cells, whereas receptor signals were enhanced (FDR<0.2) in myeloid subsets including cDC2 and CSF Mac (Fig. 4B). Notably, the strongest elevation in receptor mediated signals occurred among myeloid cells themselves, suggesting the presence of autonomous feedback activation. To explore these analyses further, we examined autonomous ligand—receptor pairs in CSF Mac and identified several upregulated pairs in RBD, including *C3*–*C3AR1*, *CD55*–*ADGRE5*, and *LGALS9*–*HAVCR2* (Tim3), which are associated with immunoregulatory functions (Fig. 4C). Additionally, we detected neurodegeneration-related signals mediated by amyloid precursor protein (*APP*–*CD74*, *APP*–*SORL1*) and prion protein (*PRNP*–*ADGRG6*). It is also known that aggregated α-synuclein can induce the activation of myeloid cells ^40^. These ligand—receptor pairs may modulate the activation state of CSF macrophages and influence the CNS microenvironment. In particular, CSF Mac may help process and clear disease-associated proteins such as α-synuclein, while simultaneously suppressing excessive inflammation via Tim3 and complement regulation as reported in microglia ^41^ (Fig. 4D). Collectively, these dynamic and comprehensive changes in cell-cell interactions appear to play a critical role in shaping the immune landscape during the prodromal stage of PD.

### Genetic prioritization of cell populations highlighted myeloid population in CSF

To determine whether integrating known genetic information with our immune cell atlas could aid in prioritizing relevant cell populations in PD, we analyzed both polygenic and rare variant components. First, we used scDRS ^42^ to integrate GWAS data associated with PD ^4^, RBD ^43^, and LBD ^44^ traits, evaluating the enrichment of polygenic signals in individual cell types (Fig. S5A,B). The analysis revealed the strongest polygenic signal in CSF Mac for LBD. Note that LBD ^44^ shares genetic risks with both PD and Alzheimer’s disease, suggesting it may not be a pure synucleinopathy. For PD, significant associations were observed in cDC2 and pDC in blood and pDC in CSF. No significant associations were detected for RBD, although polygenic signals appeared to accumulate in myeloid populations within the CSF.

We further examined the expression of PARK genes, which have been identified as causative variants in familial PD, within the CSF (Fig. S6). Although some genes, such as *PARK7* and *CHCHD2*, displayed ubiquitous expression, the overall trend indicated higher expression within myeloid cells. When focusing on CSF Mac, we observed that multiple genes, including *SNCA* (encoding α-synuclein), *PRKN*, *LRRK2*, *PINK1*, and *VPS35* were upregulated in RBD and PD. In contrast, *PARK7* and *CHCHD2* showed higher expression in controls, with reduced levels in RBD and PD, suggesting a loss-of-function role of these genes in PD. Together, these results suggest that, beyond polygenic signals, familial Parkinson’s disease variants also converge in CSF myeloid cell populations, especially in CSF Mac.

## Discussion

While emerging evidence suggests an underlying inflammatory etiology for PD as demonstrated by the presence of α-synuclein autoreactive T cells in the circulation of a proportion of patients with manifest and prodromal PD, pathological studies reveal only a minimal presence of CD8^+^ T cells in the substantia nigra of PD patients early in disease pathogenesis ^45^. To elucidate early mechanisms of disease pathogenesis in PD, we performed single-cell RNAseq analysis of paired CSF and blood from 84 subjects with different stages of PD and compared them to 15 age-matched healthy subjects and 29 patients with MS. We found a previously unrecognized pleocytosis that is most pronounced in patients with RBD, a prodromal synucleinopathy. In prodromal PD CSF, single-cell RNAseq analysis revealed increased frequencies of a population of microglia like macrophages, termed CSF Mac, exhibiting inflammatory responses characterized by increased expression of IL6-JAK-STAT3 and *TNFRSF1A* and *TNFRSF1B* signaling pathways, with a prominent lack of T cell activation. This is the opposite of what is observed in MS CSF where these is a loss of CSF Mac and increases in activated T cells. Single cell analysis of peripheral blood monocytes in in early prodromal PD revealed increased expression of ITGA4 in circulating CD16 monocytes that was confirmed by flow cytometry consistent with monocyte migration into the CNS. Thus, these findings reveal a myeloid mediated inflammatory process in the CNS of patients with prodromal PD, suggesting a novel pathologic mechanism in disease etiology of PD.

We compared immune populations with single-cell resolution in the CSF of subjects with new-onset MS and RBD. In contrast to MS CSF, where we observed a decreased frequency of CSF Mac, this macrophage population expressing microglia-like features was increased in the CSF of patients with prodromal PD, with a pronounced TNF/IL-6/JAK-STAT signature. Inhibition of the JAK/STAT pathway has been implicated in exerting protective effects in PD ^46–48^. In addition, treatment of MS with B cell depletion is 98% effective in preventing new inflammatory exacerbations; concurrently, the CSF Mac population becomes exponentially expanded in the CSF with increased secretion of TNFα by circulating monocytes ^17^. Taken together, these data suggest fundamental differences in the activity of TNFα pathways between prodromal PD and MS. Moreover, these experimental results are consistent with large-scale epidemiologic investigations of patients with IBD, demonstrating that anti-TNFα can substantially decrease the incidence of new-onset PD ^19^.

Our observations comparing PD and MS are in line with a fundamental observation of human inflammatory diseases. Specifically, the therapeutic efficacy found with blocking an inflammatory pathway in one autoimmune disease can lead to flare-ups in other autoimmune diseases. Two prominent examples include anti-IL-17 blockade that has strong efficacy in treating psoriasis but has the risk of new onset or exacerbation of IBD ^49^. Another example is anti-TNFα blockade, an effective treatment in IBD and rheumatoid arthritis that can cause exacerbations in patients with MS ^31^. These paradoxical observations are related to the different genetic architectures of autoimmune diseases where a risk haplotype in one disease can be protective in another ^28^. Thus, we hypothesized that comparing the TNFα pathways among different CNS inflammatory diseases may give insight into disease pathogenesis. However, only a randomized control investigation of patients with prodromal PD can provide definitive evidence for a role of TNFα in the pathogenesis of PD.

Compared to the significant changes in the CSF myeloid populations, relatively minor changes in T cell populations were observed in prodromal PD. Specifically, our analysis replicated previous studies ^27^ showing increases in circulating and CSF naïve CD4^+^ cells in prodromal PD and PD with reductions in the proportion of CD4^+^ Tcm cells and CD8^+^ Temra cells in CSF. Additionally, recent studies by Sulzer and co-workers demonstrated that activated α-synuclein reactive T cells in mouse models of PD cause inflammation in the gut tissue with the absence of CNS inflammation. These results are in contrast to T and B cell populations in patients with early relapsing/remitting MS, which display markedly inflammatory signals in CSF T cells and in parenchymal lesions which are relatively absent in prodromal PD. The prominent activation of CSF myeloid cells in the absence of T cell activation in prodromal PD suggest a fundamentally different mechanism of autonomous neuroinflammation mediated predominantly by the innate as opposed to the adaptive immune system. In this regard, synchronized myeloid cell activations in brain parenchymal microglia, CNS-broader macrophage are observed that are in equilibrium with human CSF Mac.

In summary, these observations in mouse models and direct examination of humans with prodromal PD leads us to hypothesize that the disease begins with activation of α-synuclein reactive T cells in the gut, perhaps related to the known microbiome dysbiosis in PD patients, leading to aggregation of α-synuclein in the intestines. It is known that α-synuclein can propagate from the gut, via the vagus nerve, to the brain ^50^, where it has been shown that α-synuclein aggregates can activate CNS border-associated macrophages ^40^ and dural myeloid cells, which are synchronized with the CSF Mac. In this model, α-synuclein–reactive T cells may act locally in the dura sinuses as suggested by mouse model and not directly in the brain parenchyma, consistent with our observations in human that in total contribute to the degeneration of CNS dopamine-producing neurons. Together, these data suggest a novel mechanism of myeloid cells as opposed to T cell-mediated autoimmune disease.

## Supplementary Material

Supplement 1

Supplement 2

Supplement 3

## Data Availability

Codes will be available on Github (https://github.com/yyoshiaki/2025_PD_PBMCCSF_scRNAseq) upon acceptance. Raw fastq files Raw fastq files will be available in the ASAP CRN cloud for the PD and RBD cohort, and dbGAP and GEO for the MS cohort upon acceptance. The processed scRNA-seq data will be available in CZ CELLxGENE upon acceptance.
