# Supplementary material for: Inflamed Microglia like Macrophages in the Central Nervous System of Prodromal Parkinson’s Disease
Source: bioRxiv. 2025 May 21:2025.05.16.654530. Preprint. [Version 1] doi: 10.1101/2025.05.16.654530 (PMC12139992; doi:10.1101/2025.05.16.654530)
Supplement: Supplement 1 [file media-1.docx]

## Materials and Methods

**Study design**

We recruited 84 subjects, including 36 individuals with Rapid Eye Movement (REM) Sleep Behavior Disorder (RBD), 15 Healthy Controls (HC), 18 individuals with Parkinson’s disease without RBD (PD), and 18 with PD and RBD (PD-RBD) between May 2021 and June 2024. We used multiple recruitment sources, including Yale research registries, HIPAA-compliant medical records searches, outreach to local sleep clinics and Facebook posts. All subjects provided written or online informed consent to participate in this study. The study and all recruitment materials received necessary approvals from the Yale Institutional Review Board.

Details of the methods for subject clinical assessments, and olfactory and biomarker testing are described in Reddy *et al*. ^1^ Briefly, for subjects with suspected prodromal PD, the diagnosis of RBD was confirmed by video polysomnography. For patients with PD who reported symptoms of RBD, a clinical history of RBD was deemed sufficient, and polysomnograms were not required. The diagnosis of PD was established based in the Movement Disorders Society PD Diagnostic Criteria. Full Inclusion/Exclusion criteria for the various diagnostic groups are shown in **Supplementary** **Table 1**. Subjects completed a comprehensive battery of clinical assessments, generally spread across 2 or 3 virtual and in-person visits over the span of one month. All subjects underwent lumbar puncture, with withdrawal of up to 30 mL of CSF while the subject was in the upright position. In addition, 150 ml of blood was drawn to support immunological and biomarker investigations. CSF of all subjects was assayed for synuclein aggregating activity (SAA) by Amprion Biosciences ^2^, and all RBD subjects underwent Dopamine Transporter Single Photon Emission Computerized Tomography (DaT-SPECT) Imaging using ^123^I – Ioflupane ^3,4^, with the data analyzed as described in Reddy et al ^1^. The clinical and biomarker assessments performed are summarized in **Supplementary** **Table 2.** Details of the clinical assessments performed and outcomes can be found in the online data supplement.

Hyposmia was defined as a UPSIT test score below the 15^th^ percentile for age and gender ^5^ ^6^. DaT Scans were interpreted as showing evidence of dopaminergic deficit if the mean striatal binding ratio (SBR) was < the 65^th^ percentile for age- and gender-matched norms ^7^. CSF samples were analyzed at Amprion for Synuclein Aggregating Activity (SAA) ^2^, and results were reported as either “detected” or “not detected”. The probability that individuals with RBD actually were manifesting prodromal PD was estimated using the online Movement Disorders Society Prodromal Parkinson Disease Calculator (<https://www.movementdisorders.org/Prodromal-PD-Calculator.htm>) based on the data acquired at study visits. For each subject a probability risk score (0-100%) was assigned. Scores were further categorized as “High” (> 80%) and “Intermediate” if 50%-79% and “Low” below 50%. A composite biomarker score was calculated based on the number of markers present (DaT SBR <U 65%ile, hyposmia, and CSF SAA status).

As for MS samples, all patients had early onset relapsing remitting MS and had not been on previous immunomodulatory treatments. A small subset of patients had received IV solumedrol within 3 months of the sample collection. Patient CSF samples were obtained for clinical diagnosis, and healthy donor CSF samples were obtained under voluntary enrollment into our research study in accordance to IRB regulations. Data from a subset of patients were previously included in our earlier publication ^8^.

**Single-cell sample processing**

Paired blood and cerebrospinal (CSF) samples were collected from each subject. Peripheral blood mononuclear cells (PBMCs) were isolated from fresh whole blood via density gradient centrifugation by layering blood diluted 1:1 in phosphate-buffered saline (PBS) over Ficoll-Paque. Red blood cells were lysed using ACK lysing buffer (Gibco) per the manufacturer’s protocol and filtered through a 35 um filter. PBMC cells were resuspended in PBS for loading into 10x Genomics Chromium at concentrations ranging from 700 to 1200 cells/uL.

CSF was kept on ice until processing, and total CSF volume was recorded. Total CSF was centrifuged at 300 x g for 10 minutes using a swing bucket centrifuge, and the supernatant was carefully aspirated, leaving about 500 uL without disturbing the cell pellet. The CSF cell pellet were resuspended and total number of CSF cells was counted using a hemocytometer (INCYTO C-Chip, DHCN015). CSF cell concentration (cells/uL) was calculated based on total CSF cell count and the original CSF volume. The resuspended CSF cell sample was then transferred to a 1.5 mL tube and centrifuged at 300 x g for 10 minutes and the final cell concentration was adjusted to 700 to 1200 cells/uL for loading into 10x Genomics Chromium platform.

**Droplet-based single-cell RNA sequencing**

For paired single-cell RNAseq, both PBMCs and CSF cell samples for the same subject were run in parallel, and single-cell libraries of gene expression, TCR and BCR were prepared using the 10x Genomics Chromium Single Cell 5’ V(D)J Reagent Kit v2 chemistry, following the the manufacturer’s protocol (10x Genomics). A target recovery of 8,000 cells per sample was set for the blood and CSF samples. The generated single-cell libraries were sequenced using Illumina NovaSeq6000 S4 at a sequencing depth of 300 million reads per sample, with an average sequencing depth of 50,000 reads per cell.

### Single-cell RNAseq analysis

Generated fastq files were aligned to human genome reference using 10x Genomics Cell Ranger (8.0.1) with the default parameters. References, refdata-gex-GRCh38-2024-A and refdata-cellranger-vdj-GRCh38-alts-ensembl-7.1.0 were downloaded at 10x Genomics official website. The single-cell data for each sample were processed using the scanpy (1.9.6) pipeline. For quality control, cells with larger than 4000 total counts or mitochondrial gene count percentage larger than 10 were removed. Then, the individual datasets were concatenated, and we further removed cells that had less than 500 genes expressed and genes with less than 50 cells expressing them. Furthermore, the doublet prediction was conducted by package Scrublet ^9^, and cells with doublet score larger than 0.15 were removed. Cells with mitochondrial genes > 20% or ribosomal genes < 5% were removed as low-quality cells. We normalized (sc.pp.normalize_total) gene expression, log-transformed it (sc.pp.log1p).

To filter out platelets and red blood cells, we excluded cells that showed normalized expression greater than 1 in any of the following genes: PPBP, GNG11, PF4, and HBB. After filtering, we performed regression to remove the effects of total counts and the percentage of mitochondrial gene expression. We then used scVI ^10^ to extract the latent space, with sample ID and 10x chemistry as categorical covariates and total_counts, pct_counts_mt, and pct_counts_ribo as continuous covariates. To compute the nearest neighbors, we used the latent space from scVI as the representation with sc.pp.neighbors. Finally, UMAP visualization was generated with sc.tl.umap. For cell type assignments, we computed clusters using Leiden algorithm (sc.tl.leiden with resolution = 1), and embedded them using UMAP algorithm (sc.tl.umap). Annotation was performed on the interactive platform, CellxGene VIP ^11^, based on Leiden clusters. Sub-cluster level preprocessing was performed using the same procedures. For the subcluster level analysis, parameters were modified depending on the population (sc.tl.leiden: resolution = 0.7-2). Cell annotation was performed on CellxGene VIP platform. For subclusters, which included doublets and low gene expression clusters, we removed them and re-embedded them. CellTypist ^12^ was used to validate clustering and annotation (model: COVID19_HumanChallenge_Blood ^13^ and Immune_All_Low ^12^. The composition analysis was performed using scCODA ^14^ with the default parameters. Differentially expressed genes (DEGs) were calculated using sc.tl.rank_genes_groups with the default parameters and visualized using EnhancedVolcano. For the visualization, genes with mean expression > 0.5 were used.

Gene set enrichment analysis was performed using decoupleR ^15^ following the official tutorial (https://decoupler-py.readthedocs.io/en/latest/notebooks/msigdb.html). Briefly, using the over-representation analysis approach, the activity of HALLMARK gene sets was calculated for each cell. The differential activity was measured by decoupler.rank_sourses_groups function (reference='HC', method='t-test_overestim_var')). Sample-wise gene set activity differences were tested using a Mann-Whitney U test and FDR was calculated using The Benjamini-Hochberg procedure.

For the comparison of CSF Mac with MS, we used the preprocessed data generated in our previous study ^16^.

### GWAS integration using scDRS

The public GWAS summary statistics were used for analysis (PD ^17^, RBD ^18^, Lewy Body Dementia (LBD) ^19^). The cohort included. Gene scores were computed using MAGMA software as described by Zhang *et al* ^20^. First, we performed single nucleotide polymorphism (SNP) annotation with gene locations (NCBI37.3, https://ctg.cncr.nl/software/MAGMA/aux_files/NCBI37.3.zip) and the reference data created from 1000 genomics Phase3 (g1000_eur, https://ctg.cncr.nl/software/MAGMA/ref_data/g1000_eur.zip) using magma --annotate (with the option, window = 10,10). Next, we calculated the gene scores from the p-values using MAGMA. We pre-processed the dataset by normalizing the total counts to the median of the total counts (scanpy.pp.normalize_total), log transformation (scanpy.pp.log1p), and imputing gene expression using MAGIC (scanpy.external.pp.magic) ^21^. Thereafter, the polygenic enrichment for each cell was evaluated using scdrs compute-score (v1.0.3, options: --flag-filter-data True --flag-raw-count False); the number of genes for each cell was used as the covariate. Group-level statistics were calculated using scdrs perform-downstream and visualized using scdrs.util.plot_group_stats.

### Integration of MS CSF dataset

We used the MS CSF datasets previously we generated ^8^ for the comparison of the CSF Mac population between MS and RBD. To perform label transfer, we used the scANVI model ^22^ implemented by scvi-tools. We followed the tutorial available at <https://docs.scvi-tools.org/en/latest/tutorials/notebooks/scrna/scarches_scvi_tools.html>. Gene set enrichment analysis was performed with the same procedure as with the PD dataset.

### Public sc/snRNAseq analysis

For PD brain snRNAseq, the preprocessed dataset was downloaded at <https://cellxgene.cziscience.com/collections/d5d0df8f-4eee-49d8-a221-a288f50a1590>. CSF Mac upregulated genes in RBD compared to HC were extracted with the following criteria: padj < 0.1, Log2 fold change > 0.2, mean expression > 0.5. For extracted microglia cells, the enrichment of CSF Mac DEGs was calculated using sc.tl.score_genes. Visualization was performed using matplotlib, seaborn, and statannotation. For mouse dura scRNAseq, we used a dataset generated by Posner et al., (Under review). The control and PD model mice have no endogenous murine αSyn due to a spontaneous deletion of the *Snca* gene but exhibit background expression of normal full-length human αSyn (BAC mouse). The PD model mice express aggregation-prone human 1-120 αSyn under the control of the tyrosine hydroxylase promoter (MI2) and develop neurological symptoms by 9-12 months. Young and old mice were used from both genotypes. To simplify the visualization, we aggregated the defined Macrophage 1 and Macrophage 2 populations as Macrophage.

### Myeloid cells integrated analysis

To compare myeloid cells across tissues, we integrated our myeloid cells data from blood and CSF with public data. Cross-tissue myeloid cell data ^12^ was downloaded at <https://cellgeni.cog.sanger.ac.uk/pan-immune/CountAdded_PIP_myeloid_object_for_cellxgene.h5ad>. For brain microglia cells, processed data was downloaded ^23^. To remove the effect of immune receptors on highly variable genes, genes related to T cell receptors and B cell receptors were removed. The retained expression was normalized (sc.pp.normalized_total with the option target_sum = 1e4) and transformed (sc.pp.log1p), and highly variable genes were assessed (sc.pp.highly_variable_genes with the options n_top_genes=3000, flavor = 'seurat_v3', batch_key = 'sample'). Cell cycle was inferred using the sc.tl.score_genes_cell_cycle function following a tutorial (https://nbviewer.jupyter.org/github/theislab/scanpy_usage/blob/master/180209_cell_cycle/cell_cycle.ipynb). The total UMI counts, percentage of mitochondrial genes, S score, and G2M score were regressed using sc.tl.regress_out and scaled using sc.tl.scale. The principal components were then computed using sc.tl.pca. The batch effect of the samples was eliminated using the Harmony algorithm ^24^. Neighbors were calculated using sc.pp.neighbors with the options n_neighbors = 20, n_pcs = 40. Cells were embedded in UMAP using sc.tl.umap.

### CD4^+^ T cell analysis

The PBMC and CSF data were processed using the pipeline developed in the previous study ^25,26^ to assign CD4^+^ T cell clusters. This pipeline employs Azimuth ^27^ for the extraction of CD4^+^ T cells and uses Symphony ^28^ for predicting CD4+ T cell clusters. For interpretability, ‘Treg Act’ has been renamed to ‘Treg Int’ from the original literature. We tested for variation in cluster frequency by modeling the per-sample cluster frequencies using a generalized linear model framework as described in the previous study ^25^. A 12-dimensional qualitative evaluation was conducted on the extracted CD4^+^ T cells using NMFproj ^25^. We applied a generalized linear model to assess feature changes per cluster as described in the previous study ^25^.

### TCR analysis

TCR analysis was performed using Scirpy ^29^. Clonotypes were defined using ir.tl.define_clonotypes (receptor_arms="all", dual_ir="primary_only"). For the assigned clones, STARTRAC ^30^ analysis was performed to infer the expansion, migration, and transition status by comparing clones in PBMC and CSF. To maintain consistent cluster definitions in both blood and CSF, we used those defined by CellTypist (Model: Immune_All_Low model).

### CCI analysis

LR analysis using CellphoneDB v5 ^31^ was performed with cell clusters in the CSF that contained at least 500 cells, employing the cpdb_statistical_analysis_method.call function with default parameters. Visualization was carried out using ktplotspy. To identify RBD-specific cell-cell interactions (CCI), we first extracted all LR pairs with p < 0.05 in Cell type A and Cell type B and selected those genes with an average expression level of at least 0.1 in the respective cell types. Next, we compared the fold changes (FC) of these genes in RBD versus HC to the FC values of genes not included in the LR-pair gene list (but still having an average expression of at least 0.1) using a Mann-Whitney U test. Finally, we performed multiple testing corrections using the Benjamini-Hochberg method (FDR). To identify RBD-specific CSF Mac interactions, we first selected LR pairs that were predicted by CellphoneDB to be significant (p < 0.05) and had average expression levels of at least 0.2 for both the ligand and receptor. From these, we extracted those pairs in which either the ligand or the receptor showed a fold change of at least 1 in RBD compared to HC. Network visualization was performed using Cytoscape ^32^.

Flow cytometry analysis

Frozen PBMCs were used for flow cytometry validation of T cells and myeloid cells. Patient peripheral blood mononuclear cells were stained with a ViaKrome 808 Fixable Viability dye following the manufacturer’s instructions. Cells were then labeled with surface antibodies for 20 min at RT. For intracellular staining, cells were fixed and permeabilized with BD Cytofix/Cytoperm Buffer (BD Biosciences) for 15 min at 4 °C, then washed with 1X BD Perm/Wash™ Buffer (BD Biosciences) or Stain buffer with BSA (BD Biosciences) ^8^. Antibody details are provided in Table S7. Cells were acquired on a Cytek Aurora Cytometer and data were analyzed with FlowJo software v.10 (Treestar). Changes in frequencies were tested using the One-way ANOVA (GraphPad Prism 10). Changes in mean fluorescence intensities (MFI) were tested using a generalized linear model with gaussian distribution and a log link function.

## Supplementary Text1

We conducted a detailed analysis of CD4^+^ T cells, which showed minor changes in PBMCs and CSF. We previously developed a pipeline for detailed analysis of circulating CD4^+^ T cells ^25^, and the same pipeline was applied to the current dataset. First, we examined frequency changes (Fig. S7A,B). The analysis showed that as previously reported, blood CD4^+^ T naive cells were increased in prodromal PD and PD. Additionally, an overall reduction in CD4^+^ Tcm cells and CD4^+^ Tem cells was observed in both blood and CSF. These increases in T naive cells and decreases in Tcm and Tem cells were validated using flow cytometry (Extended Data Fig. 8). Specifically, Tcm (Th17) cells were reduced in blood from RBD high-probabiity individuals and PD patients, as well as in CSF from PD patients. In contrast, CD4^+^ Temra (Th1) cells were increased in blood from RBD high-probability individuals and PD-RBD patients and in CSF from RBD high-probablity and PD patients. Focusing on gene programs, NMF-3 (Naive-feature or -F) was increased in CD4^+^ T naive cells in prodromal PD and PD (Fig. S9,10). In contrast, NMF2 (Th17-F) was reduced in CSF CD4^+^ Tcm (Th17) cells in PD-RBD. Broad reductions in NMF0 (Cytotoxic-F) and NMF11 (Th1-F) were observed in CD4^+^ Temra (Th1) cells in peripheral blood. Concordantly, flow cytometry analysis of CD161 (*KLRB1*), a Th17 marker gene expressed on the surface of CD4^+^ Tcm cells, revealed a decrease in its expression in PD patients (Fig. S11). In addition to transcriptomic analysis, we sought to estimate T cell activity using TCR data by STARTRAC ^30^ (Fig. S12). While no significant changes in expansion were seen between HC, RBD, or PD, a reduction in migration was observed for CD8^+^ Tem/Temra, as well as for CD4^+^ T naive/Tcm and CD4^+^ Tem/effector in PD-RBD and CD8^+^ Tem/Trm migration was reduced in PD and PD-RBD. Taken together, these results suggest that in established PD, there is an increase in CD4^+^ T naive cells, a reduction in effector functions such as Th1 and Th17, and impaired migration of T cells to the CSF. Consistent with these findings, flow cytometry analysis revealed a broad reduction of CD18 (*ITGB2*), a component of the integrin LFA-1 involved in CNS migration, in peripheral blood across CD4 Temra, CD8 Tem, and CD8 Tcm populations (Fig. S13). In conclusion, although an increase in the absolute number of subsets including memory T cells was observed in prodromal PD, transcriptomic analysis, TCR profiling, and flow cytometry did not support global T cell activation in either blood or CSF.

### Figure S1: QC metrics of scRNAseq atlas

(A) Distribution of the mean number of cells, number of genes, and % mitochondrial genes per patient in Blood and CSF. (B,C) Dot plot showing marker gene expressions across clusters in blood (B) and CSF (C). (D,E) Confusion matrix showing cluster consistencies between our annotations and clusters predicted by CellTypist ^12^ (Model: COVID19_HumanChallenge_Blood ^13^) in the blood (D) and CSF (E).

### Figure S2: Global gene activity changes

(A) Heatmap showing the number of upregulated genes in both blood (left) and CSF (right). Upregulated genes were defined using scanpy with LFC > 0.2, Padj < 0.05, and mean expression > 0.5; the RBD high-risk group is highlighted with dotted lines. (B-F) Hallmark gene sets show higher activity in RBD (D), PD (B,E), and PD-RBD (C,F) vs. HC across cell types in CSF (B,C) and blood (D-F) (Methods). Only positively associated gene sets were visualized. The dashed line at the bottom indicates Padj = 0.05. Panels include only clusters containing >1000 cells for blood and >500 cells for CSF.

### Figure S3: Similarity between CSF macrophages and myeloid cells from the whole body.

(A,B) To compare gene expression programs across the body, we integrated myeloid cells from our blood (PBMC ours) and CSF (CSF ours) samples, cross-tissue myeloid cells (Crosstissue) ^12^, and brain microglia (MicroOlah) ^23^. (A) Dot plot showing marker gene expressions. (B) Transcriptome similarities per.

### Figure S4: PD microglia and dural macrophage changes is similar to CSF Mac change.

(A,B) Brain samples from PD and HC donors were analyzed. (A) Distributions of the enrichment scores for genes upregulated in RBD CSF Mac (versus HC) within 75 PD cases and 25 unaffected control brain microglia across various brain regions (Methods). Data was downloaded from a previous report ^33^. *: p-value < 0.05. (B) Dot plot show the representative genes in PD and control brain microglia. Data was downloaded from a previous report ^33^. (C,D) Dural myeloid cell populations from human truncated αSYN expressing PD model mice (MI2BAC, labeled as truncated αSYN) and control mice (BAC, labeled as Ctrl) were analyzed. (C) UMAP plot showing the cell types and marker genes for macrophages. (D) Heatmap showing the gene expression of representative genes such as TNF-related genes and MHC class II-related genes in macrophage population.

### Figure S5: scDRS unveiled polygenic enrichment of synucleopathies in CSF myeloid cell populations

(A) Heatmaps show genetic disease associations in CSF populations evaluated using scDRS ^20^. The summary statistics from three genome-wide association studies (PD ^17^, RBD ^18^, Lewy Body Dementia (LBD) ^19^) were used. Heatmap colors depict the proportion of significant cells (FDR < 0.2). Squares denote significant disease associations (FDR < 0.05), and cross symbols denote significant heterogeneity in association (FDR < 0.05). For the analysis, small clusters (< 500 cells) were removed. (B) scDRS score distribution of UMAP embeddings.

### Figure S6: Expression of PD risk genes across CSF clusters

(A) Dot plot showing gene expression of curated PD risk genes in CSF clusters. Heatmap (right) shows the aggregated PD risk gene scores across clusters. (B) Dot plot showing PD risk genes in CSF Mac across disease conditions. The values were scaled per gene.

### Figure S7: Detailed analysis of CD4T cells

Detailed analysis of CD4^+^ T cells using a reference mapping and NMFproj ^25^. From CSF and PBMC samples, CD4^+^ T cells were extracted using Azimuth, and detailed CD4^+^ T clusters were predicted using Symphony. The 12 gene programs were calculated using NMFproj (Fig. S10,11). (A,B) Dot plot showing changes in cell frequency at cluster L2 resolution in blood (A) and CSF (B). Dot colors show coefficients, and sizes show the significance of the generalized linear model (GLM). Only significant dots (padj < 0.05) are shown.

### Figure S8: Validation of T cell frequency changes by flow cytometry.

Proportion of T cell fractions measured by flow cytometry.

### Figure S9: Gene program changes in CD4^+^ T cell subpopulations in Blood.

NMF cell features change depending on the disease condition. Dot plots depicting NMF cell feature changes in each cell type. Dot colors show coefficients, and sizes show the significance of GLM. GLM was performed with a model, NMF cell feature ~ disease. Only significant dots (padj < 0.05) are shown. The heatmaps at the top of each plot display the standardized values of the GLM intercept for each feature, representing the baseline activity of each feature in each cell.

### Figure S10: Gene program changes in CD4^+^ T cell subpopulations in CSF.

NMF cell features change depending on the disease condition. Dot plots depicting NMF cell feature changes in each cell type. Dot colors show coefficients, and sizes show the significance of GLM. GLM was performed with a model, NMF cell feature ~ disease. Only significant dots (padj < 0.05) are shown. The heatmaps at the top of each plot display the standardized values of the GLM intercept for each feature, representing the baseline activity of each feature in each cell.

### Figure S11: Loss of CD161 in CD4^+^ Tcm cells

(A) % CD161 positive cells in CD4^+^ Tcm measured by flow cytometry. (B) Dot plot showing *KLRB1* (coding CD161) expression CD4^+^ Tcm across disease condition.

### Figure S12: STARTRAC analysis revealed the global dysfunction of memory T cells in the periphery.

(A-C) Expansion (A), Transition (B), and Migration (C) inferred by STARTRAC ^30^ in blood and CSF across disease conditions (Methods). A two-tailed Welch’s T-test was performed between HC and other conditions. ns: p > 0.05, *: p <= 0.05, **: p <= 0.01

### Figure S13: Loss of *ITGB2* (CD18) in T cells.

(A) MFI of CD18 (coded by *ITGB2*) in T cell populations. (B) Dot plot showing RNA expression of *ITGB2* in T cells in blood. The values were scaled.

## Reference

1 Reddy VL, E. S., Deerhake, ME, Renal R, Benyakoub A, Mead K, Chrysotosoum C, Patel S, Sibyl JP, Koo BB, Cedarbaum JM. . Characterizing Parkinson’s Disease Clinical and Biomarker Interactions in REM Sleep Behavior Disorder *Submitted*

2 Russo, M. J. *et al.* High diagnostic performance of independent alpha-synuclein seed amplification assays for detection of early Parkinson's disease. *Acta Neuropathol Commun* **9**, 179 (2021). <https://doi.org/10.1186/s40478-021-01282-8>

3 Booij, J. *et al.* Imaging of dopamine transporters with iodine-123-FP-CIT SPECT in healthy controls and patients with Parkinson's disease. *J Nucl Med* **39**, 1879-1884 (1998).

4 Seibyl, J. P. & Kuo, P. What Is the Role of Dopamine Transporter Imaging in Parkinson Prevention Clinical Trials? *Neurology* **99**, 61-67 (2022). <https://doi.org/10.1212/WNL.0000000000200786>

5 Brumm, M. C. *et al.* Updated Percentiles for the University of Pennsylvania Smell Identification Test in Adults 50 Years of Age and Older. *Neurology* **100**, e1691-e1701 (2023). <https://doi.org/10.1212/WNL.0000000000207077>

6 Berg, D. *et al.* MDS research criteria for prodromal Parkinson's disease. *Mov Disord* **30**, 1600-1611 (2015). <https://doi.org/10.1002/mds.26431>

7 Jennings, D. *et al.* Conversion to Parkinson Disease in the PARS Hyposmic and Dopamine Transporter-Deficit Prodromal Cohort. *JAMA Neurol* **74**, 933-940 (2017). <https://doi.org/10.1001/jamaneurol.2017.0985>

8 Wei, J. *et al.* Transcriptomic profiling after B-cell depletion reveals central and peripheral immune cell changes in multiple sclerosis. *J Clin Invest* (2025). <https://doi.org/10.1172/JCI182790>

9 Wolock, S. L., Lopez, R. & Klein, A. M. Scrublet: Computational Identification of Cell Doublets in Single-Cell Transcriptomic Data. *Cell Syst* **8**, 281-291 e289 (2019). <https://doi.org/10.1016/j.cels.2018.11.005>

10 Gayoso, A. *et al.* A Python library for probabilistic analysis of single-cell omics data. *Nat Biotechnol* **40**, 163-166 (2022). <https://doi.org/10.1038/s41587-021-01206-w>

11 Li, K. *et al.* Cellxgene VIP unleashes full power of interactive visualization and integrative analysis of scRNA-seq, spatial transcriptomics, and multiome data. *bioRxiv*, 2020.2008.2028.270652 (2022). <https://doi.org/10.1101/2020.08.28.270652>

12 Dominguez Conde, C. *et al.* Cross-tissue immune cell analysis reveals tissue-specific features in humans. *Science* **376**, eabl5197 (2022). <https://doi.org/10.1126/science.abl5197>

13 Lindeboom, R. G. H. *et al.* Human SARS-CoV-2 challenge uncovers local and systemic response dynamics. *Nature* **631**, 189-198 (2024). <https://doi.org/10.1038/s41586-024-07575-x>

14 Buttner, M., Ostner, J., Muller, C. L., Theis, F. J. & Schubert, B. scCODA is a Bayesian model for compositional single-cell data analysis. *Nat Commun* **12**, 6876 (2021). <https://doi.org/10.1038/s41467-021-27150-6>

15 Badia, I. M. P. *et al.* decoupleR: ensemble of computational methods to infer biological activities from omics data. *Bioinform Adv* **2**, vbac016 (2022). <https://doi.org/10.1093/bioadv/vbac016>

16 Wei, J. *et al.* Systems Analysis of Immune Changes after B-cell Depletion in Autoimmune Multiple Sclerosis. *bioRxiv* (2024). <https://doi.org/10.1101/2024.02.07.576204>

17 Nalls, M. A. *et al.* Identification of novel risk loci, causal insights, and heritable risk for Parkinson's disease: a meta-analysis of genome-wide association studies. *Lancet Neurol* **18**, 1091-1102 (2019). <https://doi.org/10.1016/S1474-4422(19)30320-5>

18 Krohn, L. *et al.* Genome-wide association study of REM sleep behavior disorder identifies polygenic risk and brain expression effects. *Nat Commun* **13**, 7496 (2022). <https://doi.org/10.1038/s41467-022-34732-5>

19 Siderowf, A. *et al.* Assessment of heterogeneity among participants in the Parkinson's Progression Markers Initiative cohort using alpha-synuclein seed amplification: a cross-sectional study. *Lancet Neurol* **22**, 407-417 (2023). <https://doi.org/10.1016/S1474-4422(23)00109-6>

20 Zhang, M. J. *et al.* Polygenic enrichment distinguishes disease associations of individual cells in single-cell RNA-seq data. *Nat Genet* **54**, 1572-1580 (2022). <https://doi.org/10.1038/s41588-022-01167-z>

21 Yasumizu, Y. *et al.* Spatial transcriptomics elucidates medulla niche supporting germinal center response in myasthenia gravis-associated thymoma. *Cell Rep* **43**, 114677 (2024). <https://doi.org/10.1016/j.celrep.2024.114677>

22 Xu, C. *et al.* Probabilistic harmonization and annotation of single-cell transcriptomics data with deep generative models. *Mol Syst Biol* **17**, e9620 (2021). <https://doi.org/10.15252/msb.20209620>

23 Olah, M. *et al.* Single cell RNA sequencing of human microglia uncovers a subset associated with Alzheimer's disease. *Nat Commun* **11**, 6129 (2020). <https://doi.org/10.1038/s41467-020-19737-2>

24 Korsunsky, I. *et al.* Fast, sensitive and accurate integration of single-cell data with Harmony. *Nat Methods* **16**, 1289-1296 (2019). <https://doi.org/10.1038/s41592-019-0619-0>

25 Yasumizu, Y. *et al.* Single-cell transcriptome landscape of circulating CD4(+) T cell populations in autoimmune diseases. *Cell Genom* **4**, 100473 (2024). <https://doi.org/10.1016/j.xgen.2023.100473>

26 Yasumizu, Y. PBMC/CSF single-cell RNAseq CD4+ T cell reference mapping. *protocol.io* (2025). <https://doi.org/dx.doi.org/10.17504/protocols.io.q26g7mqj1gwz/v1>

27 Hao, Y. *et al.* Integrated analysis of multimodal single-cell data. *Cell* **184**, 3573-3587 e3529 (2021). <https://doi.org/10.1016/j.cell.2021.04.048>

28 Kang, J. B. *et al.* Efficient and precise single-cell reference atlas mapping with Symphony. *Nat Commun* **12**, 5890 (2021). <https://doi.org/10.1038/s41467-021-25957-x>

29 Sturm, G. *et al.* Scirpy: a Scanpy extension for analyzing single-cell T-cell receptor-sequencing data. *Bioinformatics* **36**, 4817-4818 (2020). <https://doi.org/10.1093/bioinformatics/btaa611>

30 Zhang, L. *et al.* Lineage tracking reveals dynamic relationships of T cells in colorectal cancer. *Nature* **564**, 268-272 (2018). <https://doi.org/10.1038/s41586-018-0694-x>

31 Efremova, M., Vento-Tormo, M., Teichmann, S. A. & Vento-Tormo, R. CellPhoneDB: inferring cell-cell communication from combined expression of multi-subunit ligand-receptor complexes. *Nat Protoc* **15**, 1484-1506 (2020). <https://doi.org/10.1038/s41596-020-0292-x>

32 Shannon, P. *et al.* Cytoscape: a software environment for integrated models of biomolecular interaction networks. *Genome Res* **13**, 2498-2504 (2003). <https://doi.org/10.1101/gr.1239303>

33 N, M. P. *et al.* A multi-region single nucleus transcriptomic atlas of Parkinson's disease. *Sci Data* **11**, 1274 (2024). <https://doi.org/10.1038/s41597-024-04117-y>
